# Neuromechanics of Dynamic Balance Tasks in the Presence of Perturbations

**DOI:** 10.3389/fnhum.2020.560630

**Published:** 2021-01-20

**Authors:** Victor Munoz-Martel, Alessandro Santuz, Sebastian Bohm, Adamantios Arampatzis

**Affiliations:** ^1^Department of Training and Movement Sciences, Humboldt-Universität zu Berlin, Berlin, Germany; ^2^Berlin School of Movement Science, Humboldt-Universität zu Berlin, Berlin, Germany

**Keywords:** muscle synergies, balance, balance control, postural control, balance training, neuromuscular organization

## Abstract

Understanding the neuromechanical responses to perturbations in humans may help to explain the reported improvements in stability performance and muscle strength after perturbation-based training. In this study, we investigated the effects of perturbations, induced by unstable surfaces, on the mechanical loading and the modular organization of motor control in the lower limb muscles during lunging forward and backward. Fifteen healthy adults performed 50 forward and 50 backward lunges on stable and unstable ground. Ground reaction forces, joint kinematics, and the electromyogram (EMG) of 13 lower limb muscles were recorded. We calculated the resultant joint moments and extracted muscle synergies from the stepping limb. We found sparse alterations in the resultant joint moments and EMG activity, indicating a little if any effect of perturbations on muscle mechanical loading. The time-dependent structure of the muscle synergy responsible for the stabilization of the body was modified in the perturbed lunges by a shift in the center of activity (later in the forward and earlier in the backward lunge) and a widening (in the backward lunge). Moreover, in the perturbed backward lunge, the synergy related to the body weight acceptance was not present. The found modulation of the modular organization of motor control in the unstable condition and related minor alteration in joint kinetics indicates increased control robustness that allowed the participants to maintain functionality in postural challenging settings. Triggering specific modulations in motor control to regulate robustness in the presence of perturbations may be associated with the reported benefits of perturbation-based training.

## Introduction

During daily-life activities, humans are constantly required to maintain stable locomotion in different environmental conditions that present variable and often unpredictable locomotor disturbances. Challenging balance control by using perturbations has been described as an effective intervention for reducing fall risk in different populations (Jöbges et al., 2004; Okubo et al., 2017; Hamed et al., 2018a; Mansfield et al., 2018). Compliant or unstable surfaces represent a possibility to introduce external mechanical perturbations (i.e., an alteration of the function of a biological system induced by external mechanism) and challenge balance control. Increasing the base of support (i.e., a stepping response) and counter-rotating segments around the center of mass are two of the main mechanism to recover a loss of balance (Hof et al., 2005). Previous studies of our group have reported that training these mechanisms in the presence of perturbations improves muscle strength of the lower extremities and stability performance (Arampatzis et al., 2011; Hamed et al., 2018b; Bohm et al., 2020). Nevertheless, despite the effectiveness of training in the presence of perturbations on balance performance has been generally accepted, the mechanisms explaining the improvement are still not fully understood.

Maintaining functionality despite the increased challenges induced by perturbations is a fundamental characteristic of biological systems defined as robustness (Kitano, 2004). In the presence of perturbations, the neuromuscular system overcomes challenges by modifying its control strategies in a highly coordinated and tuned manner, so that the motor task can be executed properly (Torres-Oviedo and Ting, 2007, 2010; Munoz-Martel et al., 2019). The idea that the neuromuscular system faces the redundancy of the available degrees of freedom by activating functionally related muscle groups rather than individual muscles is well accepted (Bernstein, 1967; Bizzi et al., 1991). The coordinated patterns of muscle activity are commonly known as muscle synergies and are flexibly combined to produce robust motor output (Bizzi et al., 2008; Santuz et al., 2018; Munoz-Martel et al., 2019). We have previously reported that a modulation of muscle synergies in challenging locomotion conditions allows the human system to increase the robustness of the motor control by widening the motor primitives, or time-dependent components of muscle synergies (Santuz et al., 2018, 2020a). Further, we found that the motor system generates less unstable and less complex motor primitives in the presence of perturbations, making the motor execution less prone to the influence of disturbances (Santuz et al., 2020a). In our opinion, understanding the modulations of motor control in the presence of perturbations is a key element to provide insight on the effects of external perturbations on postural control, yielding knowledge potentially useful for (a) explaining the positive effects of the perturbation-based interventions and (b) improving the design of effective exercise programs. In the presented study, we asked the participants to perform forward and backward lunges in both stable and unstable conditions to mimic one of the above mentioned balance-recovery mechanisms (i.e., increasing the base of support). The use of the muscle synergies approach might allow us to understand the organization of muscle coordination that underlies the adaptation of postural control in the presence of perturbations.

Therefore, the purpose of the current study was to investigate the effects of perturbations induced by unstable surfaces, on the mechanical loading and modular organization of motor control in the lower limb muscles during forward and backward lunges. Specifically, we investigated the spatiotemporal organization of muscle activation patterns using the muscle synergy concept and the resultant joint moments of the lower extremities during perturbed and unperturbed forward and backward lunges. We expected to find modulations of motor control in the presence of perturbations reflected in the spatiotemporal components of muscle synergies and the resultant joint moments. Specifically, we hypothesized that lunging on unstable surfaces would result in a reorganization of muscle synergies by modifying the time-dependent motor primitives and time-independent motor modules to increase the robustness of control. We also expected an increased mechanical loading of lower limb muscles.

## Materials and Methods

### Experimental Protocol

Fifteen healthy, regularly active young adults volunteered for the study (11 males, 4 females, height 1.75 ± 0.10 m, body mass 67 ± 11 kg, age 28 ± 5 years). None of the participants had a history of neuromuscular or musculoskeletal impairments, nor any injury at the time of the measurements or in the previous 6 months. The study was reviewed and approved by the Ethics Committee of the Humboldt-Universität zu Berlin (HU-KSBF-EK_2018_0013). In accordance with the Declaration of Helsinki, all the participants gave written informed consent for the experimental procedure.

**Figure 1:**
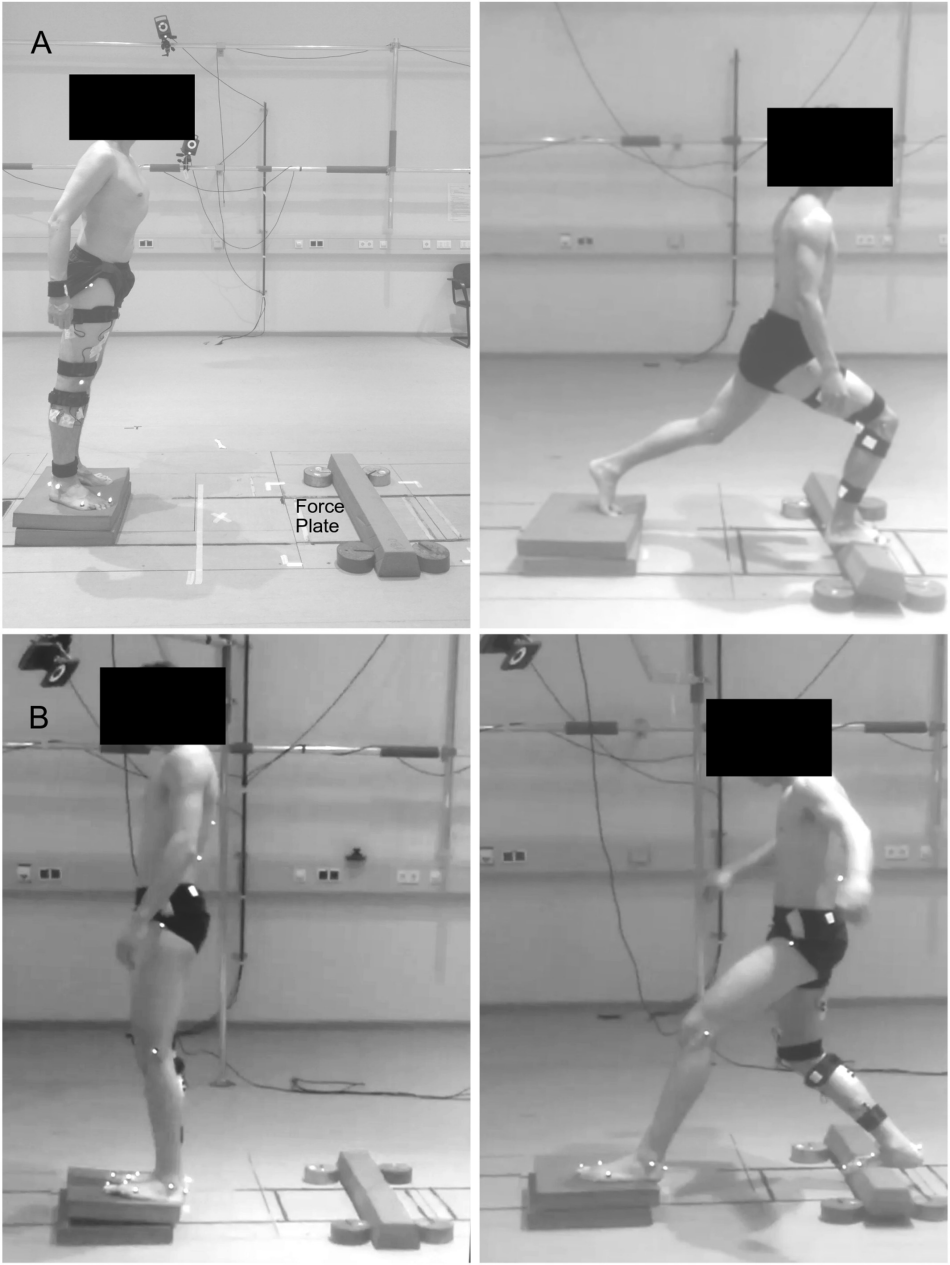
Visual description of the performed task. Participants were asked to lean forward **(A)** or backward **(B)** as far as possible, take a step reaction and hold the achieved lunge position until steady state. Fifty trials were performed on solid stable ground and 50 on foam pads, used as unstable ground condition.

Participants were asked to stand in a comfortable position and lean as far as possible until they were forced to take a step reaction with their right leg onto a target marked in the middle of a force plate (sampling frequency 1 kHz, AMTI BP600, Advanced Mechanical Technology, Inc., Watertown, MA, USA), and hold the achieved lunge position until they felt stable, i.e., steady state. The same task was performed in two directions by leaning forwards or backward (Figure 1). The starting point was set at a distance from the target equal to 70% of each participant's lower limb length (measured from the Cresta Iliaca to the Lateral Malleolus of the recovery limb) for the forward and 60% for the backward lunge. Participants performed a series of lunges on two different surfaces: hard uniform stable ground (SG) and from a foam pad (2 x Airex® Balance Pad, 50 x 41 x 6 cm, Airex Switzerland) to a foam beam (Sport-Thieme Balance beam EVA foam, 95 x 16.5 x 5.8 cm^3^, Sports-Thieme Germany) used as unstable ground (UG) to introduce external mechanical perturbations during the task. The foam beam was fixed to the force plate by double-sided tape and four five-kilogram weight disks aided to prevent a possible sliding. If the participant was not able to maintain the achieved lunge position, moved the left foot or the beam flipped or slid out of position, the attempt was considered failed and repeated. In each trial, participants performed 52 valid lunges for each direction and ground condition at a self-managed pace. The order of the trials was randomized and a self-managed rest period (minimum 3 min, seating was allowed) was given in-between trials.

A ten-infrared-camera motion capture system (Vicon Motion Systems, Oxford, U.K.) operating at 250 Hz was used to collect kinematics from 20 spherical (diameter 14 mm) reflective markers placed over the following anatomical landmarks: spinal process of the second, seventh, and tenth thoracic along with the second lumbar vertebrae, the greater trochanter, lateral and medial epicondyle of the femur, Achilles tendon insertion on the calcaneus, lateral malleolus, tip of the first toe, the dorsal margin of the first and fifth metatarsal heads. The lower limb markers were recorded bilaterally. Also, the muscle activity of the following 13 ipsilateral (right side) muscles was recorded using a 16-channel wireless electromyography (EMG) system (Myon m320, Myon AG, Schwarzenberg, Switzerland), with a sampling frequency of 1 kHz: Gluteus medius (ME), gluteus maximus (MA), tensor fasciae latae (FL), rectus femoris (RF), vastus medialis (VM), vastus lateralis (VL), semitendinosus (ST), biceps femoris (long head, BF), tibialis anterior (TA), peroneus longus (PL), gastrocnemius medialis (GM), gastrocnemius lateralis (GL), and soleus (SO). EMG and force plate analog data streams were collected together with the kinematics and then converted to digital information within the same A/D converter (Vicon MX Giganet). All further offline analysis was performed using R v 3.6.1.

### Cycle Segmentation

The interval of interest was defined as the time frame from the lift-off of the right foot until a steady-state after touchdown was achieved. The aforementioned lift-off defining the beginning of the lunge was assessed by the “foot acceleration and jerk” algorithm (Santuz et al., 2018). This approach has been previously validated using force plate data with true errors being 12 ± 18 ms for walking, −16 ± 23 ms for running and 13 ± 9 ms for the estimation of a single leg standing lift-off (means ± s.d.). First, we identified touchdown as the first non-zero value observed in the ground reaction force (GRF) data. Then, the foot kinematic data was low-pass filtered using a 4th order IIR Butterworth zero-phase filter with a cut-off frequency of 50 Hz (Maiwald et al., 2009) and the second and third derivative of the fifth metatarsal marker's position (for obtaining the acceleration and jerk, respectively) were calculated. An estimation of the lift-off (LOe, where “e” stays for “estimated”) was identified as the global maximum of the fifth metatarsal's vertical acceleration in a time window between the touchdown and 800 ms before it. Finally, the “true” lift-off was identified in a reasonably small neighborhood of the LOe (−50, 200 ms) as a characteristic minimum in the vertical acceleration (i.e., when the jerk equals zero).

The main stance phase of the recovery step ends around the maximum of knee joint flexion after touchdown. Therefore, we defined the end of the lunge as the time point with minimum fluctuation in the knee angle after maximum flexion. The minimum fluctuation was found using the technique of change point detection implemented in the function “e.divisive” from the R package ecp (James and Matteson, 2015; James et al., 2019). Briefly, the function performs nonparametric estimation of change points. A statistical significance is assigned to changes in the slope of the knee angle curve. When the slope stops to significantly depart from zero (i.e., when the knee joint is in a fixed, steady position), the beginning of the steady state is found and the cycle is trimmed at that time point.

The time window between the lift-off and the minimum fluctuation at the knee joint angle defined the duration of the cycle. Additionally, the time to achieve the minimum fluctuation from touch-down was also used to compare the performance between ground conditions. After removing the first and last cycles, all the following variables were calculated individually, cycle by cycle, and then the average of the central 50 cycles was used as a representative dataset for each participant in each direction and ground condition.

### EMG Assessments

The linear envelopes of the EMG signals were obtained by applying a 4th order IIR Butterworth zero-phase high-pass filter, full-wave rectification and low-pass filtered. Cut-off frequencies were 50 Hz (high-pass) and 20 Hz (low-pass) (Santuz et al., 2017, 2018). The amplitude was normalized to the maximum activity of each muscle for each direction. For the muscle synergies extraction, the amplitude was normalized to the maximum activation recorded for each muscle in each trial (Torres-Oviedo and Ting, 2007; Munoz-Martel et al., 2019) followed by a subtraction of the minimum activity, thus each EMG signal ranged between zero and unity. Also an EMG coactivation index was obtained by calculating the ratio between the averaged normalized EMG activities of the antagonist and the averaged agonist EMG activity for each related joint. Therefore, the corresponding ratios were calculated as follows:

Hip:

(1) $$\begin{array}{r} \frac{\left(FL+RF\right)/2}{\left(ME+MA\right)/2} \end{array}$$

Knee:

(2) $$\begin{array}{r} \frac{\left(BF+ST\right)/2}{\left(RF+VM+VL\right)/3} \end{array}$$

Ankle:

(3) $$\begin{array}{r} \frac{TA}{\left(GM+GL+SO\right)/3} \end{array}$$

Each interval of interest, from all above mentioned variables, was thereafter time-normalized to 200 points with 50 points assigned to the swing and 150 to the stance phase. The time-normalized intervals were then cut and pasted one after the other (i.e., concatenated).

### Muscle Synergies Assessment

A custom R script [R v 3.6.1 (R Core Team, 2017), R Foundation for Statistical Computing, Vienna, Austria] implementing the classical Gaussian NMF algorithm (Lee and Seung, 1999; Santuz et al., 2018) was used for extraction of the muscle synergies. The concatenated EMG data vectors were grouped in an m × n matrix V, where *m* = 13 (number of muscles) and n = number of points. This matrix was factorized such that V ≈ V_R_ = WH. Where V_R_ represents a new reconstructed matrix that approximates the original matrix V and both H and W described the synergies necessary to accomplish a movement. H represents the r × n time-dependent coefficients (motor primitives) matrix (Dominici et al., 2011; Santuz et al., 2017) of the factorization, where r represents the number of synergies necessary to reconstruct the signal and n the number of data points. W represents the m × r motor modules matrix (Gizzi et al., 2011; Santuz et al., 2017), containing the time-invariant muscle weightings. The update rules for H and W are presented as follows [Equations (4) and (5)].

(4) $$\begin{array}{r} H_{i+1}=H_{i}\frac{W_{i}^{T}V}{W_{i}^{T}W_{i}H_{i}} \end{array}$$

(5) $$\begin{array}{r} W_{i+1}=W_{i}\frac{V{\left(H_{i+1}\right)}^{T}}{{W_{i}H_{i+1}\left(H_{i+1}\right)}^{T}} \end{array}$$

The limit of convergence was defined as the amount of synergies that did not improve the reconstruction of the signals with the addition of an extra module and it was reached when a change in the calculated R^2^ between V and V_R_ was smaller than 0.01% in the last 20 iterations (Cheung, 2005; Santuz et al., 2017, 2018). This was done for a number of synergies successively increased from 1 to a maximum of the rounded 75% of the number of assessed muscles (i.e., 10 synergies) (Santuz et al., 2019). The computation was repeated 10 times for each synergy, each time creating new randomized initial matrices H and W, in order to avoid local minima (d'Avella and Bizzi, 2005). For each of the 10 synergies the solution with the highest R^2^ was then selected.

The minimum number of synergies required to reconstruct the original EMG signals was chosen using a linear regression model fitting the curve of R^2^ values vs. synergies for all the synergies. The mean squared error was then repeatedly calculated, each time removing the lower synergy point, until only two points were left or until the mean squared error fell below 10^−5^ (Santuz et al., 2017, 2018), assuming that at this point the addition of an extra synergy did not improve the quality of the reconstruction. In order to compare the extracted synergies and give them a functionally meaningful interpretation, we classified the extracted synergies using an unsupervised method, previously described in detail (Santuz et al., 2020b). Unsupervised algorithms reduce possible operator-dependent bias in the classification. The algorithm clustered the primitives that showed similar shapes. Fundamental primitives, i.e., primitives that show one peak in their activation pattern (Santuz et al., 2017, 2018), were then ordered based on their center of activity [CoA, see Equations (6)–(8)]. The primitives that were not clustered, were classified as combined (i.e., two or more fundamental synergies blended into one). In our data, combined synergies usually constitute 10–30% of the total extracted synergies. Due to the lack of consensus in the literature on how to interpret them, for the combined synergies we did not calculate the metrics reported in the following paragraphs.

### Metrics for Comparison of Motor Primitives

The motor primitives in both conditions were compared by means of the CoA and full width at half maximum (FWHM). The CoA was defined as the angle of the vector (in polar coordinates) that points to the center of mass of that circular distribution (Cappellini et al., 2016; Santuz et al., 2018). The polar direction represented the cycle's phase, with angle 0 ≤ θt ≤ 2π. The CoA is defined by the following equations:

(6) $$\begin{array}{r} A=\overset{n}{\underset{t\;=\;1}{\sum }}\left(\cos\theta_{t}\times H_{t}\right) \end{array}$$

(7) $$\begin{array}{r} B=\overset{n}{\underset{t\;=\;1}{\sum }}\left(\sin\theta_{t}\times H_{t}\right) \end{array}$$

(8) $$\begin{array}{r} CoA=arctan\left(B/A\right) \end{array}$$

### Kinematics and Resultant Joint Moments

The kinematics of the hip, knee and ankle joint were calculated from the 3D trajectories of the stepping limb using a custom R v 3.6.1 algorithm. Subsequently, the resultant joint moments for the aforementioned joints were calculated using an inverse dynamics procedure (Arampatzis et al., 1999) with segmental masses and inertial parameters derived from literature (Winter, 2005). Similar to the EMG data, kinematics and resultant moments from each region of interest for each cycle were subsequently time-normalized to 200 points, with 50 points attributed to the swing phase and 150 points to the stance phase.

### Statistical Analysis

We performed a statistical parametric mapping (SPM) paired *t*-test (Pataky, 2010; Pataky et al., 2013) between conditions on the following time-dependent variables: sagittal plane kinematics and joint resultant moments, the Euclidean norm of the GRF, EMG activity of each muscle and coactivation ratios. A critical threshold t^*^ was calculated based on the temporal smoothness of the input data through Random Field Theory and a test statistics SPM{t} was evaluated at each point in the time series. In the case that SPM{t} exceeded t^*^, a significant difference was detected. Significance level was set at 0.05 and Bonferroni corrected for multiple comparisons (*N* = 3 for joints, *N* = 13 for EMG of muscles). All SPM calculations were performed in MATLAB using the open-source package spm1d (v 0.4.5).

To account for a possible effect of repetition on the neuromuscular organization, we split the CoA and FWHM datasets in two groups. Each group contained the first and last 25 repetitions (“early” and “late” cycles, respectively). We performed a two-way ANOVA for repeated measures on the CoA and FWHM with ground (stable, unstable) and repetition (early and late cycles) as within subject factors. A Tukey *post-hoc* analysis with false discovery rate α-value adjustment was conducted in the case of a significant interaction between the factors. To investigate differences in the motor modules between conditions, the same statistical approach was performed using muscles and ground condition as independent variables. All the significance levels were set to α = 0.05 and analyses were conducted in R v 3.6.1.

## Results

### Temporal Parameters

Participants needed a significantly longer time to reach a steady state after landing onto the unstable ground in the forward lunge compared to the stable condition (SG: 0.818 ± 0.210 s, UG: 1.055 ± 0.311 s, *t*(14) = −5.04; *p* < 0.001). This led to an increased duration of the task (SG: 1.117 ± 0.214 s, UG: 1.368 ± 0.319 s, *t*(14) = −5.28; *p* < 0.001). In the backward lunge, there were no statistically significant differences in the time to reach steady state between stable and unstable ground conditions [SG: 0.779 ± 0.226 s, UG: 0.863 ± 0.178 s, *t*(14) = −1.49; *p* = 0.160]. Similarly, the duration of the task in the backward lunge did not show any statistically significant differences between the two conditions [SG: 1.082 ± 0.22 s, UG: 1.188 ± 0.18 s, *t*(14) = −1.97; *p* = 0.07].

### Kinematics and Kinetics

**Figure 2:**
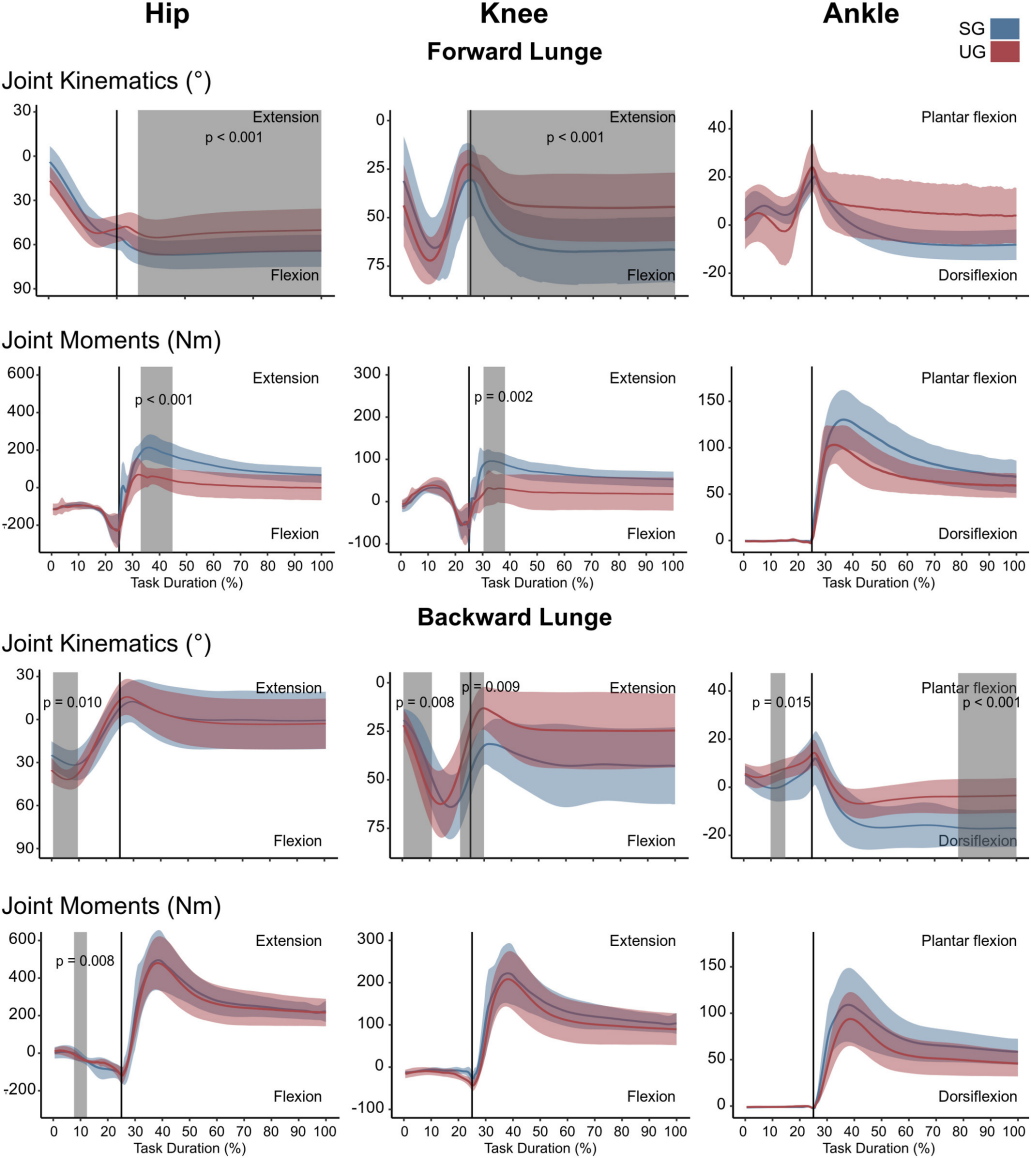
Lower limb kinematics and resultant joint moments of the forward and backward lunges from lift-off until steady state. Each panel shows the mean values and standard deviation bands for the hip, knee and ankle joint angles and moments for the stable (SG—blue) and unstable ground (UG—red) condition. Panels are presented in a time normalized base, vertical lines represent touchdown. Gray bands denote periods of significant differences assessed with a statistical parametrical mapping difference estimator Bonferroni corrected.

In the forward lunge onto the unstable ground, the hip was significantly less flexed (t^*^ = 3.989, *p* = 0.015) closely after touchdown (~30% of the lunge duration) and during most of the stabilization phase (~40–100 % of the lunge duration, t^*^ = 3.989, *p* < 0.001, Figure 2). Furthermore, the knee joint was also less flexed from briefly before touchdown and during the whole stance phase (~25–100% of the lunge duration, t^*^ = 4.177, *p* < 0.001, Figure 2). The ankle joint angle showed no differences between stable and unstable ground conditions (Figure 2). In the backward lunge, a significantly higher flexion at the hip (t^*^ = 4.182, *p* = 0.010) and knee (t^*^ = 4.181, *p* = 0.008) joint was observed at the beginning of the swing phase (first 10% of the lunge) in the unstable ground. This condition induced a lower knee flexion around the touchdown (~20–30% of the task, t^*^ = 4.181, *p* = 0.009) and ~70% of the stabilization phase (t^*^ = 4.181, *p* < 0.001, Figure 2). A lower dorsiflexion was also observed in the middle of the swing phase (~15% of the lunge duration, t^*^ = 4.074, *p* = 0.015) and toward the end of the stabilization (~85–100% of the task, *p* < 0.001) in the unstable ground.

**Figure 3:**
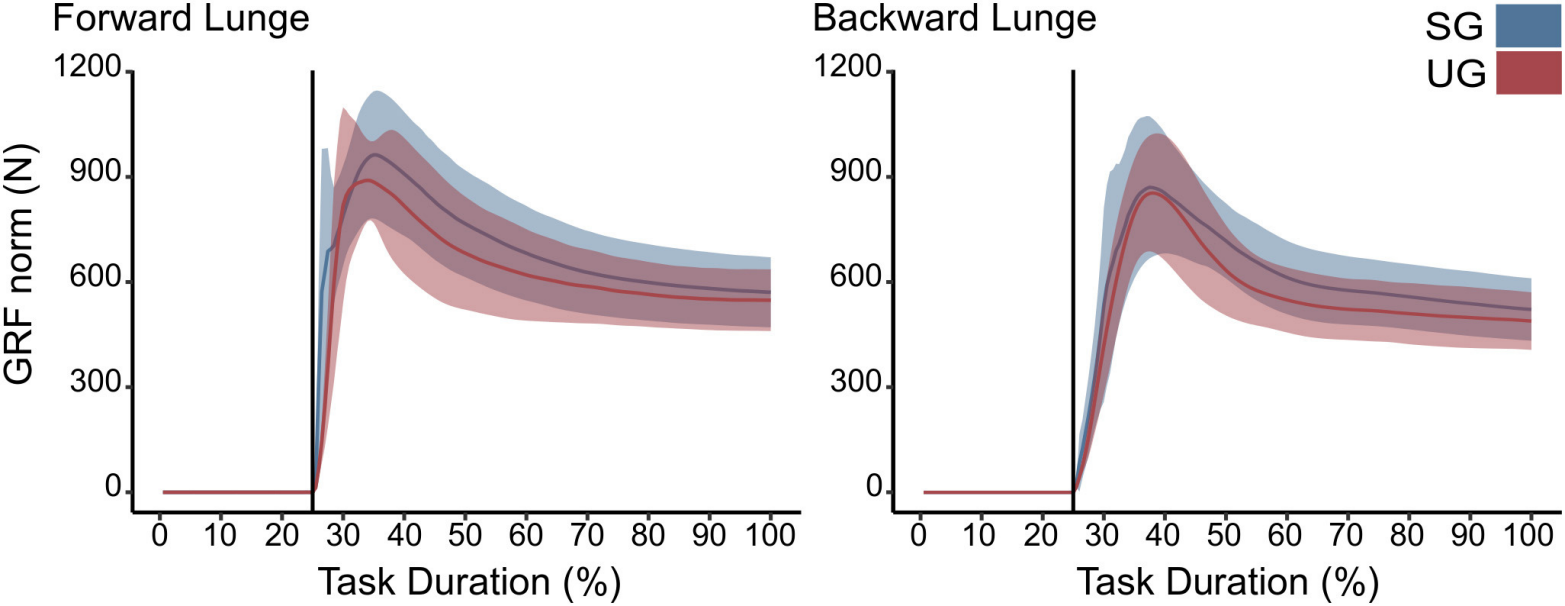
Euclidean norm of the ground reaction force (GRF) during forward and backward lunges from lift-off until steady state. Each panel show the mean values and standard deviation bands for the stable (SG—blue) and unstable ground conditions (UG—red). Both panels are presented in a time normalized base, vertical lines represent touchdown.

There were no differences in either of the lunge directions in the GRF (Figure 3). In the majority of the lunge duration, the resultant joint moments did not show statistically significant differences between the two ground conditions (Figure 2). A short decrease was found in the extensor moment at the beginning of the stabilization phase (~30–40% of the task duration) in both the hip (t^*^ = 5.427, *p* < 0.001) and knee joint (t^*^ = 5.421, *p* = 0.002) in the unstable ground during the forward lunge (Figure 2). Similarly, during the backward lunge, a brief decrease in the hip joint (t^*^ = 4.661, *p* = 0.008) was observed in the middle of the swing phase (~10% of the task duration) in the unstable ground (Figure 2).

### Electromyographic Activity

**Figure 4:**
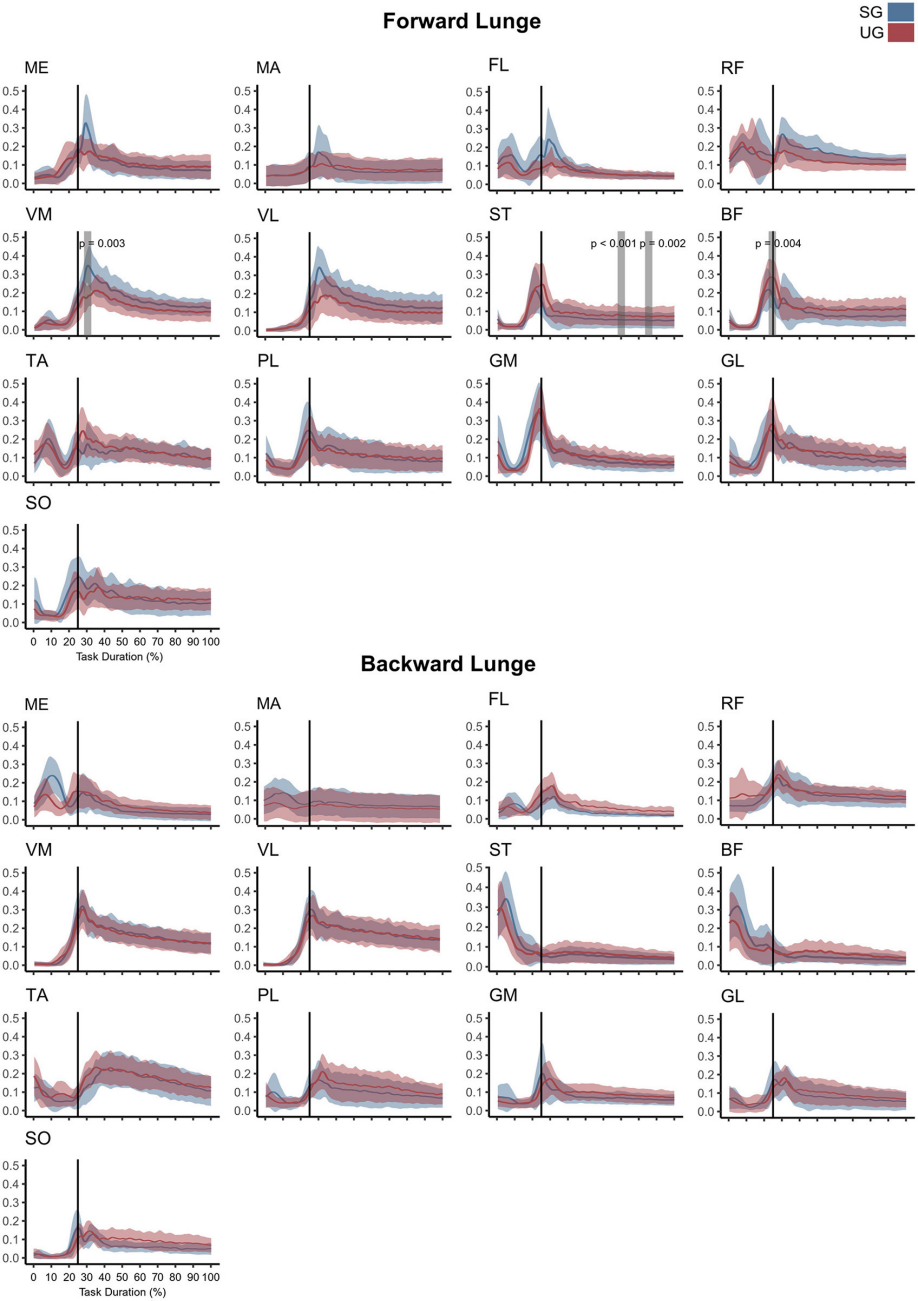
Mean values and standard deviation bands for the EMG activities for a forward and backward lunge from lift-off until steady state on stable (SG—blue) and unstable ground condition (UG—red) normalized to the maximum activity between trials. Panels are presented in a time normalized base, vertical lines represent touchdown. Gray bands denote periods of significant difference estimated with a Bonferroni corrected statistical parametrical mapping difference estimator. Muscles: Gluteus Medius (ME), Gluteus Maximus (MA), Tensor Fascia Latae (FL), Rectus Femoris (RF), Vastus Medialis (VM), Vastus Lateralis (VL), Semitendinosus (ST), Biceps Femoris (long head, BF), Tibialis Anterior (TA), Peroneus Longus (PL), Gastrocnemius Medialis (GM), Gastrocnemius Lateralis (GL), and Soleus (SO).

**Figure 5:**
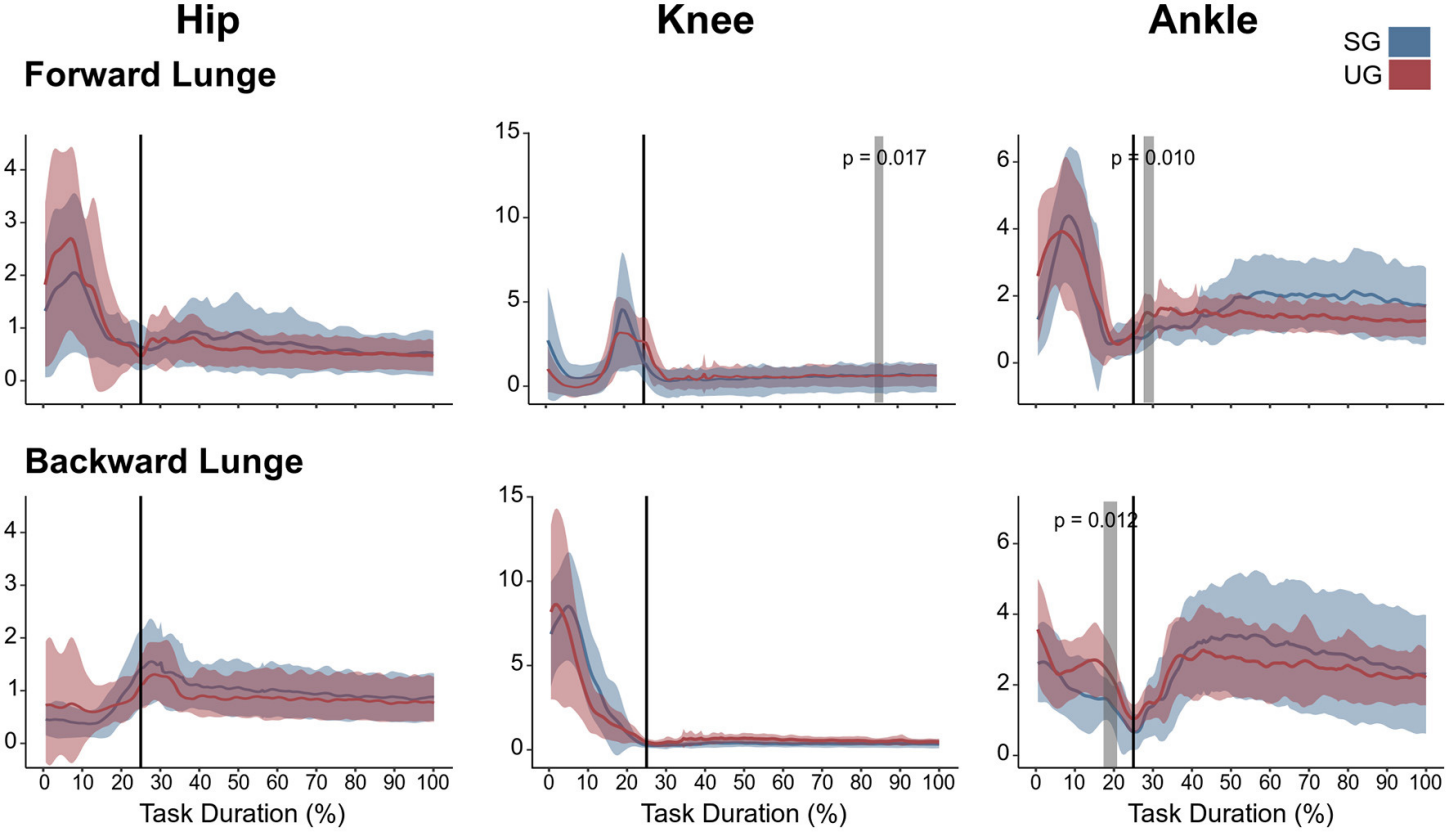
Lower limb coactivation ratios (antagonist mean/agonist mean) of the forward and backward lunges from lift-off until steady state. Each panel show the mean values and standard deviation bands for the hip, knee and ankle joint coactivation index for the stable (SG—blue) and unstable ground condition (UG—red). Gray bands denote significant differences from the statistical parametrical mapping difference estimator. Both panels are presented in a time normalized base, vertical lines represent touchdown.

Similar to the resultant joint moments, the observed significant differences in the EMG activity were short in time and not consistent (Figure 4). In the forward lunge, there was a decrease in the VM_EMG_ activity after touchdown in the unstable ground (~30% of lunge duration, t^*^ = 6.182, *p* = 0.003). The ST_EMG_ activity increased on the unstable ground around the 70% of the lunge duration (t^*^ = 6.323, *p* < 0.001) and close to the end of the stabilization phase (~90% of the lunge duration, *p* = 0.002). Similarly, BF also showed a higher EMG activity around the touchdown in the unstable condition (~25% of the lunge duration, t^*^ = 6.075, *p* = 0.004, Figure 4). In the backward lunge, a decrease in ME (t^*^ = 6.123, *p* < 0.001) and BF EMG activity (t^*^ = 5.994, *p* < 0.001) during the swing phase (~10–20% of the lunge duration, Figure 4) was observed in the unstable ground. The coactivation ratios showed also brief differences between the stable and unstable ground (Figure 5). In the forward lunge, the ratio in the unstable ground was higher briefly around the 80% of the task for the knee (t^*^ = 5.371, *p* = 0.017) and after touchdown for the ankle (~30% of the lunge duration, t^*^ = 5.1818, *p* = 0.010). In the backward lunge, the coactivation ratio in the unstable ground also increased in the ankle joint before touchdown (~25% of the lunge duration, t^*^ = 5.058, *p* = 0.012, Figure 5).

### Muscle Synergies

**Figure 6:**
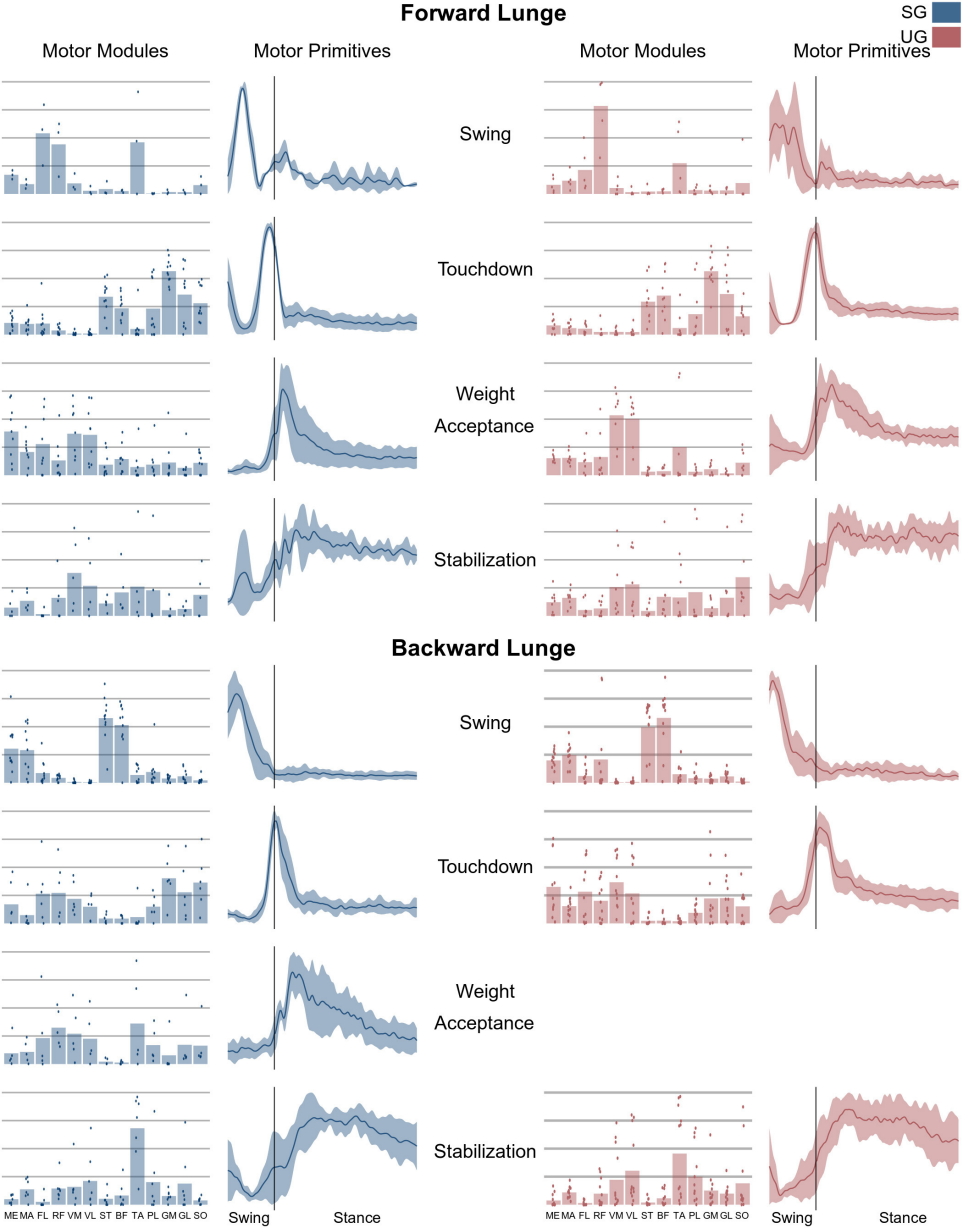
Average motor modules and motor primitives of the fundamental synergies needed to perform the forward and backward lunges on stable and unstable ground condition. The motor modules are presented on a normalized y-axis base: each muscle contribution within one synergy can range from 0 to 1 and each point represents an individual trial. Motor primitives mean and standard deviation bands are presented for one trial (from lift-off to steady-state), time-normalized to 200 points (x-axis), and amplitude-normalized to maximum (y-axis). The vertical line in the primitive panels indicates the touchdown (i.e., the beginning of the double leg stance).

The average number of synergies extracted to sufficiently reconstruct the original EMG activity was not significantly different between ground conditions in either the forward (SG = 5.0 ± 0.6, UG = 5.3 ± 0.6, *p* = 0.165) nor the backward lunges (SG = 4.7 ± 0.5, UG = 4.8 ± 0.6, *p* = 0.721). The classification identified a total of four fundamental synergies (i.e., a synergy whose motor primitive shows a single peak of activation (Santuz et al., 2018) in the forward lunge (Figure 6) on both stable and unstable ground. In the backward lunge, the recognized fundamental synergies were four in the stable and three in the unstable condition (Figure 6).

**Figure 7:**
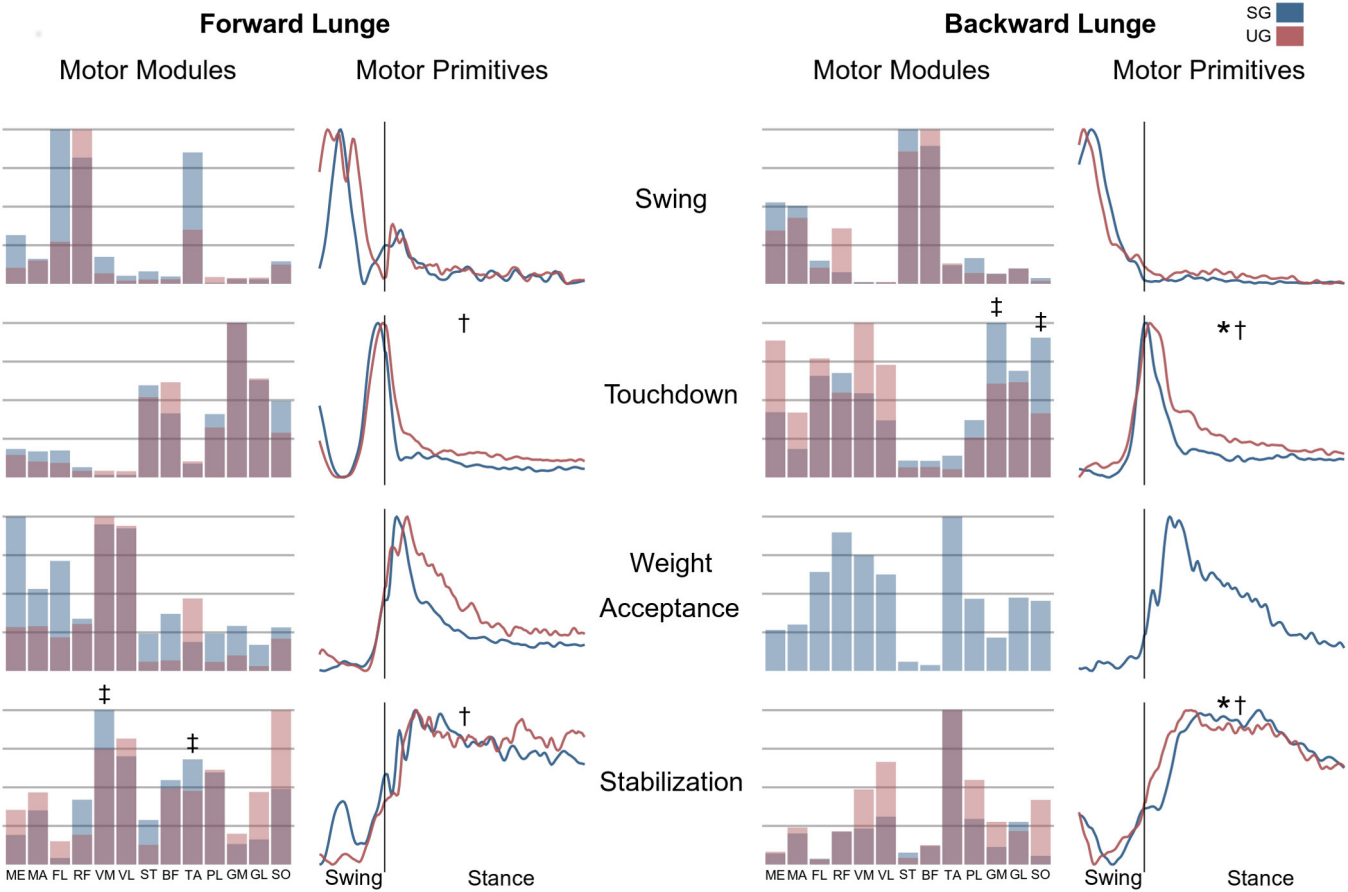
Differences for motor modules and motor primitives for the recognized synergies in the forward and backward lunges. Significant differences in the full width at half maximum of the motor primitives are denoted by asterisks (*), differences in the motor primitives center of activity by daggers (†). Double daggers (‡) denote *post-hoc* individual muscles differences in the motor modules.

**Figure 8:**
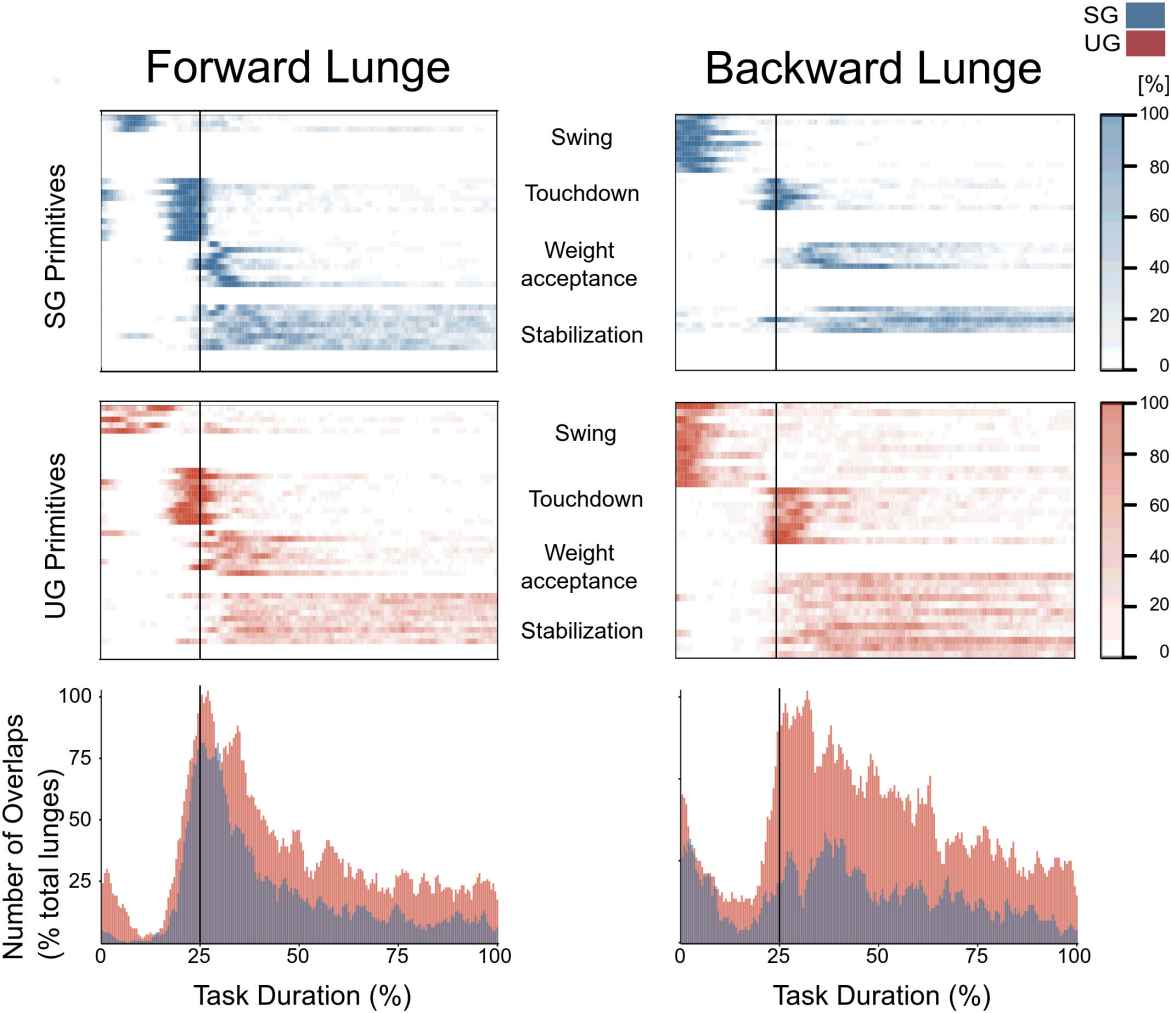
Overlapping time intervals of motor primitives for the forward (left) and backward (right) lunges on stable (SG, top-panel blue) and unstable ground (UG, middle panel red). Each row of the heat maps represents a single motor primitive. A colored time-point indicates the primitive is exceeding half maximum. Darker colors indicate higher number of occurrence across all cycles per participant. At the bottom panel the average number of overlaps across all trials and all participants per ground condition. For all graphs the x-axis full scale represents one trial (from lift-off to steady-state), time-normalized to 200 points. The vertical line indicates the touchdown i.e., the beginning of the double leg stance.

In both the forward and backward lunges, the fundamental muscle synergies were associated with temporally different phases of the task. The first synergy was related to the swing phase and in the forward lunge showed a major involvement of the foot dorsiflexors, hip flexors, and hip abductors. In the backward lunge, the main contribution in the swing synergy resulted from the hamstrings and glutei. The second synergy identified the touchdown phase with a main contribution of the plantar flexors and hamstrings in the forward lunge, and plantar flexors, knee extensors, hip flexors and abductors in the backward lunge. The third synergy was functionally associated with the acceptance of the body weight and, in the forward lunge, was characterized by a main activity of knee and hip extensors. In the backward lunge, the third synergy was recognized only in the stable ground and showed a main contribution of knee extensors, dorsiflexors, and plantar flexors (Figure 6). The fourth synergy reflected the stabilization phase of the task and was, in the forward lunge, characterized by the involvement of dorsiflexors, knee extensors, lateral foot stabilizers, and plantar flexors, whilst in the backward lunge the dominant contribution of the stabilization synergy corresponded to dorsiflexors (Figure 6). In the forward lunges we observed a significant effect of the ground type (Table 1). The CoA was significantly shifted to a later moment in the touchdown [*F*_(1, 20)_ = 13.43, *p* = 0.004, Table 1] and stabilization [*F*_(1, 12)_ = 15.31, *p* = 0.004, Table 1] synergies in the unstable compared to stable condition. The motor module of the stabilization synergy showed also a statistically significant ground effect [*F*_(1, 14)_ = 11.84, *p* = 0.001, Table 1], the *post-hoc* analysis revealed a significant reduction in the contribution of VM (*p* = 0.001) and TA over unstable ground (*p* = 0.020, Figure 7). Moreover, we found a significant repetition effect, resulting in a significant shift toward an earlier CoA of the stabilization synergy [*F*_(1, 20)_ = 5.21, *p* = 0.004, Table 1] and a decreased FWHM in the swing synergy [*F*_(1, 18)_ = 20.75, *p* = 0.010, Table 1]. An interaction of ground and repetition was observed in the CoA of the stabilization synergy [*F*_(1, 12)_ = 7.425, *p* = 0.026, Table 1]. The *post-hoc* analysis revealed that the CoA shifted significantly later in time in the second half of the trial on SG (*p* = 0.016), and in both early and late cycles on UG (*p* < 0.001) compared to the early cycles on SG. Also, the *post-hoc* indicated that the CoA of the late cycles on SG was earlier than the CoA of the early cycles on UG (*p* < 0.001). Lunging backward on unstable ground resulted in a significant modification of the touchdown and stabilization synergies. The touchdown primitive shifted its CoA toward after the touchdown [*F*_(1, 30)_ = 6.507, *p* = 0.016, Table 1] and increased its FWHM [*F*_(1, 30)_ = 4.974, *p* = 0.033, Table 1]. Furthermore, the motor module of the touchdown was also modified by the unstable ground [*F*_(1, 14)_ = 4.11, *p* = 0.44, Table 1] with a significant lower contribution of GM (*p* = 0.009) and SO (*p* = 0.002) compared to the stable ground (Figure 7). In addition, the stabilization primitive was also wider [*F*_(1, 34)_ = 8.945, *p* = 0.005, Table 1] and shifted earlier in time [*F*_(1, 34)_ = 8.408, *p* = 0.007, Table 1] on UG. Lunging on UG resulted in an increased number of overlaps from shortly before the touchdown and through the entire stance phase of the lunge in both directions. In the forward lunge also an increased number of overlaps were observed at the beginning of the swing phase. This phenomenon resulted from a larger number of overlaps between the touchdown and weight acceptance as well as the weight acceptance with the stabilization motor primitives in the UG condition (Figure 8). Considering the absence of the weight acceptance primitive in the backward lunge in UG, the increased overlapping was observed between the touchdown and stabilization motor primitives (Figure 8).

**Table 1:** Differences for Full Width at Half Maximum (FWHM) and Center of Activity (CoA) for each extracted synergy between conditions (SG = stable ground, UG = unstable ground) and repetition (Early and Late cycles) for forward and backward lunges.

**Synergy**	**SG**		**UG**		***p*****-values**		
	**Early**	**Late**	**Early**	**Late**	**Ground**	**Repetition**	**Interaction**
**FORWARD LUNGE**							
**FWHM**							
Swing	33.3 ± 8.0	23.4 ± 5.9	29.8 ± 13.5	27.8 ± 12.6	0.935	0.010*	0.088
Touchdown	21.8 ± 3.1	20.5 ± 3.6	21.1 ± 5.1	22.9 ± 5.0	0.623	0.790	0.162
Weight Acceptance	21.8 ± 10.0	18.7 ± 11.0	23.2 ± 4.1	23.01 ± 5.4	0.410	0.282	0.242
Stabilization	50.9 ± 10.6	49.2 ± 14.9	45.8 ± 20.0	43.0 ± 17.6	0.252	0.378	0.901
**CoA**							
Swing	63.5 ± 44.0	44.3 ± 24.9	55.5 ± 55.6	36.0 ± 21.0	0.523	0.337	0.98
Touchdown	51.6 ± 13.5	44.8 ± 6.7	60.6 ± 10.9	56.5 ± 8.9	0.004*	0.045*	0.551
Weight Acceptance	75.9 ± 12.1	75.5 ± 9.2	80.7 ± 13.1	77.4 ± 13.8	0.468	0.173	0.293
Stabilization	109.4 ± 10.6	117.1 ± 5.5	124.3 ± 6.2	120.8 ± 6.7	0.004*	0.250	0.026*
**BACKWARD LUNGE**							
**FWHM**							
Swing	21.2 ± 5.2	20.3 ± 5.8	21.7 ± 5.8	21.1 ± 7.26	0.718	0.663	0.944
Touchdown	18.8 ± 5.3	20.7 ± 5.0	29.0 ± 8.2	26.0 ± 13.4	0.033*	0.702	0.483
Weight Acceptance	36.0 ± 17.2	27.2 ± 13.4	n.a.	n.a.	n.a.	n.a.	n.a.
Stabilization	43.1 ± 22.9	46.7 ± 25.1	66.4 ± 15.6	64.8 ± 20.6	0.005*	0.807	0.714
**CoA**							
Swing	32.5 ± 21.5	29.1 ± 27.9	41.2 ± 18.6	50.9 ± 29.8	0.066	0.674	0.421
Touchdown	65.5 ± 6.2	60.3 ± 5.6	74.5 ± 11.8	69.9 ± 11.6	0.016*	0.178	0.930
Weight Acceptance	94.7 ± 14.6	94.6 ± 19.4	n.a.	n.a.	n.a.	n.a.	n.a.
Stabilization	120.1 ± 11.3	133.2 ± 11.7	112.8 ± 14.4	112.2 ± 17.2	0.006*	0.352	0.166

*Values are presented in mean ± standard deviation for each ground condition and repetition group*.

Asterisks (*) denote significant differences.

## Discussion

The purpose of the current study was to investigate the modular organization of the motor control system and the mechanical loading of the lower limb's muscles whilst lunging forward and backward in the presence of perturbations, induced by an unstable surface. We hypothesized that participants would modulate the spatiotemporal organization of their muscle synergies to cope with the unstable condition and maintain functionality by increasing their EMG activity and resultant joint moments. We found relevant modifications in the spatiotemporal structure of the muscle synergies, especially in the stabilization synergy, partly confirming our hypothesis. However, the EMG activities and resultant joint moments showed only small and inconsistent alterations.

An explicit modification in the kinematics of the lower limb was observed when lunging on unstable ground. During the forward lunge, both the hip and knee joints remained in a less flexed position compared to that achieved in the stable condition, indicating a lower range of flexion during the stance phase. Correspondingly to our findings, several studies have reported a reduced range of motion in the leg joints when interacting with soft surfaces during jumping (Prieske et al., 2015), running (Karamanidis et al., 2006), hopping (Farley et al., 1998), and landing (Hollville et al., 2020). These results have been proposed to reflect a mechanism for increasing the stiffness of the limb in order to compensate for the changes in the stiffness of the ground, allowing the system to move similarly on different surfaces (Ferris and Farley, 1997; Farley et al., 1998).

We expected an increase in the EMG activity of the leg muscles as well as adjustments in the resultant joint moments to compensate for balance control in the presence of perturbations. However, despite some brief alterations in the resultant joint moments and the EMG activity on unstable ground, these parameters behaved quite similarly between the two ground conditions. Coactivation has the potential to increase the muscle mechanical loading without modifying the resultant joint moments. However, our results showed no differences in the coactivation ratios between ground conditions that could explain the increase in muscle force after perturbation-based training reported elsewhere (Hamed et al., 2018a; Bohm et al., 2020). Nonetheless, the resultant joint moments of both forward and backward lunges can be interpreted as high. We detected maximal joint moments between 100 and 500 Nm, which are substantially higher compared to the joint moments reported for postural swaying (Hess et al., 2006) and equivalent to running (Stefanyshyn and Nigg, 1998; Arampatzis et al., 1999), jumping (Nikolaidou et al., 2017), and landing (Walsh et al., 2011; Werkhausen et al., 2018). Recently, we found greater movement instability and higher EMG activity of the leg muscles in unstable ground conditions during postural tracking of an oscillating visual target (Patikas et al., 2019). The higher activation of the leg muscles was the consequence of the increased movement instability during postural swaying on unstable ground and was interpreted as a compensation mechanism to ensure balance in the presence of external perturbations (Patikas et al., 2019). Whilst postural tracking of an oscillating visual target is a periodic movement condition and required submaximal muscle force generation, forward and backward lunges are aperiodic, high-intensity tasks. Several studies examining EMG activity in aperiodic, high-intensity landing and jumping tasks reported no relevant alterations (Prieske et al., 2013; Hollville et al., 2020) or even a downregulation of muscle EMG activity (Lesinski et al., 2017; Helm et al., 2019) on unstable compared to stable surfaces.

Lunging relies mainly on predictive control until the first 30–90 ms after touchdown (Patla, 2003). This is especially true for the present study, where participants performed several times the two lunges and, therefore were experienced about the task and characteristics of the surfaces. Furthermore, the task execution was performed with open eyes, thus based on the visual feedback information. Both, the available knowledge from experience about the intended movement and the visual input guidance, influences the motor output through predictive motor control strategies (Patla, 2003; Bierbaum et al., 2010; Bohm et al., 2012). However, the time from touchdown until steady state was on average >800 ms and therefore reactive feedback-based control components were included in the execution of the task, particularly because it was impossible to fully predict the behavior of the surface and, thus the perturbation itself. Our findings and the additional literature reports indicate that the effects of unstable surfaces on muscle EMG activity are inconsistent, intensity- and task-specific. It follows that external perturbations do not necessarily increase muscle activation. The small and inconsistent differences in the resultant joint moments, muscle EMG activity, and muscle co-activation between stable and unstable surfaces indicate a more or less similar mechanical loading in leg muscles. Earlier randomized control studies (Hamed et al., 2018a; Bohm et al., 2020) found improvements in muscle strength by exercising mechanisms of dynamic stability as forward and backward lunges in unstable conditions. In those studies, it was assumed that training on unstable surfaces that continuously introduce disturbances can increase muscle mechanical loading in the lower extremities and, thus, muscle strength (Hamed et al., 2018a). Our current study evidenced that muscle mechanical loading is not affected by unstable conditions during forward and backward lunges and this finding may be of particular interest when planning perturbation-based balance training programs.

During the execution of the investigated task, the participants reached their individual “limit of stability” (i.e., lean as far as possible) to trigger a step reaction. The main goal of the task was to keep balance after the lunge reaction (i.e., regaining the extrapolated center of mass within the base of support (Arampatzis et al., 2008). Four fundamental synergies were recognized for each lunge direction on stable ground, each of them associated to sub-functions of the lunge. The spatiotemporal structure of the synergies was modified in the unstable ground condition. In the forward lunge, the CoA of the touchdown and stabilization primitives were shifted later in time, toward the middle of the stance phase. In the backward lunge, the motor primitives of the touchdown and stabilization synergies became wider and, whilst the CoA of the touchdown primitive shifted to a later time, the CoA of the stabilization primitive shifted to an earlier time, resulting in an increased number of overlaps when lunging on UG. A temporal overlapping between chronologically-adjacent synergies might be a compensatory mechanism adopted by the CNS to cope with the postural instability resulting from disturbances (Martino et al., 2015; Santuz et al., 2018; Munoz-Martel et al., 2019; Janshen et al., 2020). Moreover, the increased overlap of the muscle synergies might create a “buffer” of motor control, enhancing the robustness of the motor system to cope with the perturbations (Santuz et al., 2018, 2019, 2020a; Janshen et al., 2020). The absence of the weight acceptance synergy during the backward lunges resulted in a reduction of the number of synergies by merging the weight acceptance and stabilization synergies in the unstable condition. Merging of synergies has been reported in stroke patients and was found to be associated with the pathology related severity (Clark et al., 2010; Cheung et al., 2012). Although it is difficult, using the current methodology, to identify the concrete neurophysiological origin of this phenomenon, it has been suggested that the merging of synergies may be an alternative solution for stroke patients to compensate the pathology-related impairments when executing a motor task (Cheung et al., 2012; Ting et al., 2015). We found also differences in the motor modules indicating modifications in the contribution of individual muscles within the synergies. These findings characterize a modulation of motor control in the unstable condition to ensure functional movement execution, less prone to disturbances. All participants were able to perform both forward and backward lunges in the unstable condition, indicating retention of functionality despite external perturbations. Muscle synergies represent modules of spinal and supraspinal interactions coordinated to create a functional motor output (Bizzi et al., 2008; Bizzi and Cheung, 2013; Ting et al., 2015) and modifications in their spatiotemporal activation patterns enhance the ability of the motor system to modulate effective robustness in challenging settings, ensuring functionality (Santuz et al., 2018, 2020a; Munoz-Martel et al., 2019).

The FWHM was not affected by the ground condition whilst lunging forwards. However, whilst lunging backwards, where visual feedback is more limited, the touchdown and stabilization primitive increased the FWHM. Widening of motor primitives is associated with challenging locomotion and interpreted as a neuromotor mechanism robustly regulating motor output in the presence of external (e.g., mechanical) (Santuz et al., 2018, 2020a) or internal (e.g., pathology-related) perturbations (Martino et al., 2014; Janshen et al., 2020). Recently, we found similar modifications in muscle synergies in wild-type mice but not in genetically modified mice that lacked feedback from proprioceptors (Santuz et al., 2019), evidencing a relevant contribution of sensory feedback in the modulation of motor control in the presence of perturbations. In the weight acceptance and stabilization synergies, sensory processing was likely involved in the motor control processes to increase the chance of reactive adjustments, based on proprioceptive information received during and after touchdown. The main alterations in the modular control occurred in the stabilization synergy. This synergy is characterized by a wide motor primitive which is extended during the whole stance phase when the participants deal with the perturbations. The observed alterations in the motor primitives of the stabilization synergy, and the shift of the touchdown CoA on both directions toward a later time after the touchdown, in the UG, indicate reactive adjustments in the modular organization as a consequence of the external perturbations. We cannot exclude any predictive or anticipatory motor control in this synergy because the participants expected mechanical disturbances after touchdown. The absence of the weight acceptance synergy in the backward lunge is likely the consequence of proactive adjustments. However, the effects of the unstable condition on the temporal components of the synergies, strongly indicate that part of the resulting perturbations were unpredictable and initiated reactive modulation of motor control to perform the task successfully.

The temporal activation pattern of the swing synergy did not show any differences between the stable and unstable conditions in both tasks. The first synergy is functionally responsible for the increase of the base of support after stability is lost to recover the extrapolated center of mass within the base of support and the second synergy functionally prepares for the contact of the leg with the ground. After the loss of balance, an increase in the base of support to regain the extrapolated center of mass within its limits is a basic postural mechanism (Arampatzis et al., 2008; Bierbaum et al., 2013) independent of the landing surface. It can be argued, that relevant components of predictive and anticipatory control during the swing phase with minor reactive adjustments may explain the similar temporal organization of the first synergies. An effect of repetitions was observed only in the swing and touchdown synergies of the forward lunge (on FWHM and CoA respectively), in both cases in the direction of a reduction of the metric. This might indicate an acute adaptation to the repeated exposure to the perturbation, thus indicating the possibility of a “learning effect.” We have to mention that in our analysis we did not consider the contralateral limb and trunk muscles that might be relevant for the stabilization process. During a step reaction, the supporting limb has been described for playing a role during the push-off phase, particularly providing time for correct positioning of the stepping leg (Pijnappels et al., 2004; Walsh et al., 2011). The stepping limb, on the other hand, is of paramount importance for both, the swing phase (Aragão et al., 2011; Arampatzis et al., 2011) and decelerating the center of mass after the touchdown (Pijnappels et al., 2004; Karamanidis and Arampatzis, 2007; Karamanidis et al., 2008; Mademli et al., 2008).

In summary, our results evidenced that the neuromuscular system adjusts its modular organization in both forward and backward lunges in the presence of perturbations. Modifying the spatiotemporal structure of muscle synergies and kinematics allowed the participants to maintain functionality in challenging settings with minor alterations of movement kinetics. The observed alterations indicate that both proactive, as well as reactive control mechanisms, were involved in the modulation of muscle synergies to regulate motor control in unstable ground conditions. Such modifications in regulating motor function in challenging settings might affect the ability of the motor system to modulate effective control robustness in response to environmental changes and may contribute to the reported stability improvements after perturbation-based exercise (Hamed et al., 2018a; Bohm et al., 2020).

## Data Availability Statement

The raw data supporting the conclusions of this article will be made available by the authors, without undue reservation.

## Ethics Statement

The studies involving human participants were reviewed and approved by Ethics Committee of the Humboldt-Universität zu Berlin (HU-KSBF-EK_2018_0013). The patients/participants provided their written informed consent to participate in this study. Written informed consent was obtained from the individual(s) for the publication of any potentially identifiable images or data included in this article.

## Author Contributions

VM-M and AA designed the experiment. VM-M conducted the experiment and analyzed the data. AS and AA substantially contributed to data analysis. VM-M, AS, and AA interpreted the data and drafted the manuscript. SB made important intellectual contributions during revision. All authors approved the final version of the manuscript and agree to be accountable for the content of the work.

## Conflict of Interest

The authors declare that the research was conducted in the absence of any commercial or financial relationships that could be construed as a potential conflict of interest.
